# The Book World of Medicine and Science

**Published:** 1895-02-09

**Authors:** 


### THE BOOK WORLD OF MEDICINE AND SCIENCE.

THE THEORY AND PRACTICE OF MEDICINE. By FREDERICK T. ROBERTS, M.D., F.R.C.P. (London: H. K. Lewis. 1894. Ninth edition. pp. 1,168. Price 21s.)

A ninth edition does not require much reviewing; the public has long since pronounced its verdict, and the long series of students brought up on Roberts' Medicine who have, year after year, entered the profession are testimony to its having fulfilled the purpose for which it was written. Not that the book as it now stands is a mere reprint. The growth of a standard text-book is a process both of development and emendation; new subjects come into prominence and require incorporation, old ones fade, and thus we find that in this edition not only has much been re-written and a good deal added, but that it has been necessary to alter in some degree the arrangement of the work. It will perhaps be a surprise to some, living as we are in the hum and buzz of the various theories and speculations of bacteriology, to find such small prominence given to that subject as a whole—although under the different diseases, notably diphtheria and tuberculosis, certain useful details are inserted. No doubt if the writer of a standard work is to be limited to what one may call standard knowledge, positive facts, the bearings of which are not likely to be disturbed within a few years, Dr. Roberts is right to limit himself to what is known, and not to enter too largely into details regarding so very fluctuating a science as bacteriology. Still it is a loss. As a cause of inflammation, for example, micro-organisms are mentioned, but the paragraph on suppuration reads strangely without any reference to them. An interesting feature in this edition is the insertion of sections on general therapeutics of the different organs in the chapters relating to their diseases. The descriptions of the various diseases and morbid conditions treated of are admirable, and the book is arranged in such an orderly manner that it is very easy to find any information which may be required. The work before us will be found a satisfactory guide to the student, and a desirable companion to the practitioner, who will find in it many helpful suggestions on many topics. It is especially as an orderly and systematic presentment of what is known in the very wide domains of general medicine that it has gained and still retains its high place in current medical literature.

POST-NASAL GROWTHS. By CHARLES A. PARKER, Assistant Surgeon to the Hospital for Diseases of the Throat, Golden Square, London. (London: H. K. Lewis. 1894. Octavo, 98 pages. Price 4s. 6d.)

This is a small work on the affection usually called "adenoids." It deals with the pathology, etiology, symptoms, and treatment of this very common affection, traversing familiar ground and not adding materially to our knowledge. The two points upon which the author chiefly insists are—first, even in cases of obstruction to nasal respiration, such as is caused by post-nasal adenoids, the breathing is nasal and not buccal during sleep, although the mouth may be and indeed is widely open; and secondly, that many of the serious ill-effects of adenoids are due to the diminished air-tension behind nasal obstruction. His discussion of the causation of snoring leaves a good deal to be desired.

PRACTICAL PHYSIOLOGY OF PLANTS. By FRANCIS DARWIN, M.A., F.R.S., and E. HAMILTON ACTON, M.A. (Cambridge: At the University Press. 1894.)

Amongst the natural science manuals issued by the Cambridge University Press, that one dealing with the physiology of plants, by Darwin and Acton, will certainly take a high position. The want of such a book has long been felt in this country by students and teachers alike, for Detmer's "Pflanzenphysiologie" scarcely meets the demands of the present day, and moreover, being in German, many are debarred from using it. The volume before us is divided into two parts, of which the first is concerned more particularly with physiological experiments, whilst Part II. is of a more advanced character, dealing with the organic constituents of plants. This branch of the subject is especially beset with difficulties, but in a very careful appendix some of the probable pitfalls are pointed out. There are a few singular misprints in the book, as *e.g.*, on p. 111, the consistent use of copper *sulphide* instead of *sulphate*. These are, however, but slight defects, and do not detract seriously from the value of the book as a whole, which will certainly become indispensable wherever botany is seriously taught.

POCKET THERAPEUTIC NOTES. (Ferris and Co., Chemists, Bristol. Second edition.)

Messrs. Ferris's new edition of their list of drugs and preparations has been brought well up to date, and is a useful little reference work. Messrs. Ferris's price being attached to each article mentioned renders the list a useful one for doctors dispensing their own medicines. The use and usual dose is mentioned in all cases, and the list of diseases at the end of the small volume is supplied with a list of medicines frequently adopted.

